# Neurodevelopmental Treatment in Children With Cerebral Palsy: A Review of the Literature

**DOI:** 10.7759/cureus.50389

**Published:** 2023-12-12

**Authors:** Sandeep Khanna, Ranganathan Arunmozhi, Chanan Goyal

**Affiliations:** 1 Physiotherapy, PhD Program, Sardar Bhagwan Singh University, Dehradun, IND; 2 Physiotherapy, Latika Roy Memorial Foundation, Dehradun, IND; 3 Physiotherapy, Sardar Bhagwan Singh University, Dehradun, IND; 4 Physiotherapy, Government Physiotherapy College, Raipur, IND; 5 Neuro Physiotherapy, Datta Meghe Institute of Higher Education and Research, Wardha, IND

**Keywords:** cp, pediatric physiotherapy, treatment of cerebral palsy, ndt, icf, cerebral palsy (cp), participation, neurodevelopmental treatment

## Abstract

This review aimed to explore the current literature on neurodevelopmental treatment (NDT) in children with cerebral palsy (CP). It also sought to determine what outcome measures are used to analyze the effect of NDT and whether these parameters are in line with the components of the International Classification of Functioning, Disability and Health (ICF). The studies published in the English language between 2000 and 2023 were included based on a search of the databases PEDro, PubMed, and Google Scholar. Studies that examined the effect of NDT on children with CP were included.

We found a total of 54 studies describing the effect of NDT in children with CP and these were included in this literature review. NDT in children with CP was found to have positive outcomes in 41 studies, while 13 studies had contradictory conclusions. Based on our findings, NDT is widely used for the rehabilitation of children with CP globally. The parameters used to assess the improvement mostly included gross motor function, balance, and postural control. The outcome measures used in studies are usually linked to body structure and function or activities domain of the ICF model by the World Health Organization (WHO). However, there is a scarcity of studies on the effect of NDT on participation, which should be the outcome of any rehabilitation program. There is scope for future research to demonstrate the effect of NDT on the participation of children with CP. Further studies with larger sample sizes and homogenous groups are recommended.

## Introduction and background

In India, 3.8% of the population has some form of disability. The most prevalent motor impairment in children is cerebral palsy (CP). CP affects approximately one-fifth of physically challenged children. The global prevalence of CP is 1.5-3.8 in 1000 births [1]. The prevalence of CP among live births in India is estimated to be about three per 1000 [2]. CP refers to a disorder of movement and posture that limits activities and is believed to be caused by nonprogressive damage to the developing brain in the early years of life [3]. This condition has a complex presentation in terms of interaction between different domains of a child’s development including motor, sensory, cognitive, linguistic, executive, and behavioral components. The variables that put one at greater risk of CP may include congenital defects of the brain, genetic predisposition, infection during pregnancy, multiple pregnancies, ischemic hypoxic encephalopathy, stroke in utero or during birth, kernicterus, low birth weight, newborn seizures, newborn meningitis or sepsis, traumatic brain damage after birth, and preterm delivery [4]. Attaining motor milestones and improvement in function in children with CP depends on various factors, including the severity of the condition, timely and appropriate intervention, parent empowerment, involvement of the family, and opportunities provided to children to practice movements in their natural environment. Globally, developmental professionals work in multidisciplinary teams to bring positive change and enhance the quality of life of children with CP and their families.

Neurofunctional approaches for the management of neurological disorders include the Affolter approach, Brunnstrom approach, proprioceptive neuromuscular facilitation devised by Dr. Herman Kabat, sensory stimulation for activation and inhibition developed by Margaret Rood, constraint-induced movement therapy, mirror therapy, task-oriented approach, and neurodevelopmental therapy/Bobath approach [5]. One of the most popular interventions among pediatric physiotherapists for managing CP globally is neurodevelopmental treatment (NDT). NDT is a problem-solving method for assessing and treating the functional limitations of individuals with CP [6]. This literature review aims to summarize available evidence of the outcome of NDT in children with CP. It also delineates the parameters and outcome measures used to analyze the change in children with CP. Another objective is to find if the outcome measures used to investigate the effect of NDT in children with CP fit with the International Classification of Functioning, Disability and Health (ICF) as NDT lays stress on improving function and participation.

## Review

Sah et al. conducted a study in 2019 involving 44 children aged 7-15 years with spastic diplegic CP. Task-oriented activity-based NDT was found to be more effective than conventional physiotherapy (PT) in improving control of the trunk, balance, and gross motor function in children diagnosed with CP. The outcome measures employed to study the effects of therapy plans were gross motor function measure (GMFM), postural assessment scale, pediatric balance scale, and trunk impairment scale [7]. Another study published in 2018 showed that an NDT-based eight-week training program increased the functional level of motor ability and independence in 15 children with hemiparetic and diparetic CP, whose ages ranged from 5 to 15 years, by improving balance and postural control. Outcome measures used in this study were GMFM, seated postural control measure, pediatric balance scale, modified timed up and go test, functional independence measure, and one-minute walking test [8].

A systematic review based on the ICF framework was undertaken in 2012 to assess the efficacy of concept-based approaches and supplementary therapies utilized in the therapy of lower limbs for children with CP. The outcomes of these studies were assessed using a configuration suggested by the American Academy for Cerebral Palsy and Developmental Medicine. NDT and functional training on gross motor function were found to have level II evidence. Besides, level IV evidence for NDT on all ICF levels was found in this study [9]. A study published in 2017 concluded that intensive NDT significantly increased gross motor function; 42 children with developmental delay and CP and developmental delay without CP were provided intensive NDT for three months followed by conventional NDT for another three months. All children in the study showed improvement in their gross motor abilities with intensive NDT. GMFM was used as an outcome measure and intensive NDT was recommended for all children with developmental delays [10].

A clinical trial published in Developmental Medicine and Child Neurology in 2004 involving children with CP found that rigorous NDT was beneficial in enhancing gross motor function. The trial involved a total of 34 children with levels of gross motor function classification ranging between 1 and 3. The outcome measure used in this study was GMFM [11]. A case series published in 2021 reported that intensive NDT had positive results in six children with neurologic conditions. six children aged 2-10 years were included in this study and the outcome measure used was GMFM-66 [12]. In a 2015 study, Labaf et al. found that NDT enhanced gross motor function in children with CP in four categories: lying and rolling, sitting, crawling and kneeling, and standing; 28 CP children were included in this study and the outcome measure used was GMFM [13].

Another study in 2017 suggested that NDT when administered to low-birth-weight preterm babies in a neonatal intensive care unit was found to lead to developmental improvement in the selected group; 96 premature children were included in the study and the outcome measure used was the test of infant movement performance [14]. A pilot study published in 2015 was designed to analyze the effect of just one session on sit-to-stand movements in CP children. Eight children aged four to six years with CP were included in this study. The findings indicated that children with CP can stand from a seated posture without employing abnormal movement patterns after attending just one NDT session [15].

The findings of a clinical series investigation revealed a remarkable improvement in visual attention due to improved motor control skills following the NDT session compared to the control session; 10 children aged 6-16 years participated in this study. The outcome measure used was the Conners Kiddie Continuous Performance Test (K-CPT) [16]. The NDT program is more beneficial than conventional therapy for children having CP. The NDT strategy enhances posture and function used in day-to-day context in children with CP. CP children aged three to nine years were selected. The outcome measure used was GMFM [17]. In an Indian study, children with CP showed a substantial increase in gross motor ability after three months of NDT [18].

A clinical trial conducted in 2022 tried to verify the activation of muscle using electromyography when using NDT in children with severe CP; 39 spastic quadriparesis CP children of similar age and same gender were included in the study. The study concluded that there was activation in multifidus, gluteus medius, rectus abdominis, and erector spinae while children were being treated with NDT [19]. A study conducted in Nigeria in 2013 aimed to find the levels of disability of CP clients receiving PT management; 30 participants aged one to six years were observed after three, six, and 12 months of undergoing NDT. It was determined that NDT PT effectively managed CP, and intervention frequency and duration were significant determinants [20].

Forty ambulatory children with CP took part in a six-week trial that aimed to examine how their walking changed after receiving NDT. Gait parameters such as stride and step length, foot angle, base of support, cadence, and velocity were measured using pedographs. Significant gains in stride and step length, foot angle, and velocity were observed in all participants [21]. A study by Turker et al. in 2015 aimed to analyze the effect of goal-directed NDT (GD-NDT) on children with CP in terms of daily living activities and motor function; 26 children aged between 5 and 17 years with CP were included in the study. They were provided GD-NDT for 12 weeks, three times per week. Positive outcomes were seen in gross motor function, health-related quality of life (HRQOL), and level of independence in the daily life of children with CP [22].

A study in 2018 aimed to determine if a 36-month-old child with bilateral spastic CP would benefit from PT intervention using neurodevelopmental strategies in line with the Bobath concept. The patient showed favorable results after the study in all ICF components, with activities being the most significantly improved component [23]. A study conducted by Besios et al. sought to assess the NDT method's efficiency in treating CP in young patients. Twenty children with cerebral palsy participated in an eight-week NDT intervention program. The study found substantial evidence that the NDT (Bobath) approach improves children's mobility [24]. The purpose of the study done by Knox and Evans in 2002 was to assess the functional benefits of NDT in CP children; 15 kids with CP diagnoses with ages ranging from 2 to 12 years were enlisted. This study showed that improvements in motor function and self-care were achieved in this population after a course of NDT [25]. A systematic review and meta-analysis performed in 2020 found that when balance-training therapies were paired with NDT, there was a significant beneficial effect on postural control. Seven different studies with a total of 194 candidates were included in this review [26].

Karabay et al. observed that, in addition to NDT, four weeks of kinesio taping (KT) or neuromuscular electrical stimulation (NMES) is beneficial in reducing kyphosis and improving sitting posture; 75 children participated in this study. The outcome measure used was the sitting section of GMFM and kyphosis levels [27]. Another study by Elbasan et al. suggested that the use of KT and NMES as an adjunct to NDT enhanced gross motor function, posture in sitting, and control of posture; 45 children aged 5-12 years participated in the study spanning six weeks. The outcome measures used were manual muscle testing, shortness tests, gross motor function classification system (GMFCS), the sitting component of GMFM, modified functional reach test, functional independence measure (WeeFIM), cerebral palsy quality of life (CP QOL), and seated postural control measurement (SCPM) [28]. A longitudinal intervention that was carried out for four years showed that conventional treatment when combined with NDT improved children's speech from incoherent to an acceptable level of functional speech [29].

New ways of providing therapy combined with NDT have shown positive outcomes in children with CP. In children having milder forms of CP, NDT when combined with video games based on Wii Fit balance was found to show both static and performance-related balance metrics improvement [30]. In 2021, Acar et al. studied the effects of NDT on eating, swallowing, and difficulty in feeding in children with CP. They concluded that in children with CP, there is an association between oral motor capabilities and control in the trunk. The eating ability of children in the group receiving NDT along with oro-motor intervention and caregiver training showed better improvement than the control group; 40 children were included in the study and the outcome measure used was the trunk impairment scale, schedule for oro-motor assessment, and pediatric quality of life inventory [31].

NDT used along with transcranial direct current stimulation (tDCS) was found beneficial for children with CP in reducing spasticity and enhancing motor development; 24 CP children were included in this study and the outcome measures used were GMFM-88, box and block test, and modified Ashworth scale [32]. In 2021, a study by Avcil et al. concluded that NDT and video game-based therapy had similar positive benefits on grip strength and functional abilities [33]. In children with CP, stabilization exercises of the trunk and neck used along with NDT resulted in improved communication, production of speech, daily activities, and quality of life [34]. A randomized controlled trial (RCT) in 2021 suggested that extracorporeal shock wave therapy given to paraspinal muscles offers significant additive value when paired with NDT in improving balance and postural control in children with hemiplegic CP. Thirty-two CP children were included in the study and the tools used were the trunk control measurement scale, timed up and go, pediatric balance scale, and trunk impairment scale [35].

A study in 2012 concluded that the short-term effects of an NDT method are more pronounced in achieving set goals post-Botox for children with CP than those of a traditional PT regime [36]. Lee et al. reported that NDT along with steadily increasing functional coaching in children with spastic CP can increase muscle thickness of leg muscles and improve motor function [37]. Vestibular stimulation combined with NDT has shown positive outcomes and hence is considered a beneficial adjunct to increase motor function [38]. In children with CP, a hip radiography follow-up program combined with NDT and a posture management program were found to reduce the natural course of hip dislocation [39]. A comprehensive approach for intervention in children with hemiplegic CP including NDT as one of the intervention methods demonstrated improvement in postural symmetry in sitting and standing [40]. Incorporating NDT concepts into a modified constraint-induced movement therapy (CIMT) protocol may be a useful intervention for children with hemiplegia to maximize functional motor skill acquisition [41]. An RCT by Kolit and Ekici determined that cognitive orientation to the daily occupational performance approach along with NDT showed clinically better outcomes than NDT alone [42].

A pilot study conducted in 2022 revealed positive outcomes in terms of balance rehabilitation when using a combination of Vojta and NDT in children with CP [43]. A study in Iran involving 22 participants with spastic CP in 2010 indicated improvement in four areas of GMFM when intervention was offered using NDT and sensory integration therapy [44]. An analysis done in 2014 by Behzadi et al. concluded that the traditional NDT and the home program Bobath approach where parents were involved in goal-making and conducting exercises at home was more beneficial when compared to traditional NDT alone. Thirty children with CP aged 0-2 years were included in the analysis and GMFM was used to measure the difference between their condition pre and post-intervention [45]. Research done in 2007 showed that intensive conventional PT including NDT along with partial body weight treadmill training (PBWTT) was more beneficial for improving the motor and ambulatory abilities of children with CP. Five children aged between two years and three months and nine years and seven months were included in the study [46]. Choi et al. concluded in their study in 2011 that both NDT and task-oriented training led to improvement in sitting posture in children with CP [47].

In 2015, Dewar et al. performed a systematic review to analyze which exercises improved control of posture in CP children. Out of the 13 exercises assessed, only five showed a medium level of proof that they were beneficial in improving postural control. The evidence for NDT benefitting control of posture in children with CP was inadequate or contradictory [48]. Another systematic review in 2019 by Zanon et al. reviewed RCTs to analyze the contrast between NDT and conventional PT for CP children. This study concluded that the effects of NDT on children with CP are unknown; further research is required to find out more about its safety and efficacy. NDT usage based on current data did not support routine use of NDT in practice [49].

In 2019, a review was conducted to summarize the effect of PT interventions on children with CP; 34 systematic reviews were included and 15 different ways of therapy were found. NDT was found to be ineffective in this study [50]. Park and Kim conducted research in 2017 to assess changes in strength, stiffness, and gross motor function in children with spastic CP after receiving NDT-based intervention. The findings suggested that this intervention was beneficial in lowering spasticity in children with CP but did not enhance gross motor function [51]. A study was conducted in 2016 on 20 CP children with GMFCS levels 1 and 2. It found Adeli suit treatment and NDT to be more successful than NDT alone in enhancing spatiotemporal gait metrics but had no significant effect on gross motor and balance [52]. A systematic review in 2008 reported that RCTs provide primarily limited information on the effectiveness of most PT techniques due to methodological limitations and variability in population, interventions, and outcomes. Well-designed trials are required, especially to analyze concentrated PT therapies including NDT [53].

To prove that postural control therapies for children with CP are helpful, more research with stronger designs is needed, as per a study by Harris et al. [54]. The motor learning coaching treatment was found more effective than NDT in terms of functional performance and retention of motor function [55]. A meta-analysis in 2022 by Velde et al. recommended the de-implementation of NDT in children with CP as interventions for improving motor function based on activities and body structure and function were found more effective than NDT [56]. A single-blinded randomized controlled study involving 18 children with CP showed positive outcomes with the use of modified pilates exercises on control of posture, walking, trunk, and balance as compared to NDT [57].

Research conducted in 2019 indicated that respiratory exercises with NDT were more beneficial in increasing respiratory function when compared to only conventional NDT in spastic quadriplegic CP children. Thirty children were included in the study and lung volumes were compared to show outcomes [58]. A systematic review was conducted by Martin et al. in 2010 to identify the common PT ways used in school-aged children with CP and the evidence supporting them was critically reviewed. Strong evidence was found in favor of strengthening the targeted muscle groups, and new data emerged to support functional training. Concerning treadmill training, NDT, and appropriate dosage of PT, further high-level evidence is needed [59].

A 2009 study tried to look into how modified Adeli suit therapy (MAST) affected the gross motor skills of children with CP; 36 children with CP participated. Intervention was provided to the participants two hours per day for five days per week for four weeks. When it came to helping children with CP improve their gross motor function, the MAST was superior to either the Adeli suit therapy or the NDT [60]. A paper summarized the effects of various physical interventions used for children with CP. It stressed the need to do more research to analyze the effect of physical interventions on function and disability and not only on impairments [61]. A study was conducted by Furtado et al. in 2022 to find and evaluate published papers on physical therapy in children and adolescents from Brazil with CP by using the ICF framework. One of the interventions being used was NDT. Among the studies, the components of intervention did not look into participation, and, in the assessed outcomes, only 1.1% related to participation [62].

In previous studies, numerous parameters were used to indicate improvement with the use of NDT in children with CP. These areas of improvement are summarized in Table 1.

**Table 1:** Areas of improvement observed in various studies assessing the effect of NDT on children with CP NDT: neurodevelopmental treatment; CP: cerebral palsy

S. no	Area of improvement using NDT
1	Gross motor [7,10,11,12,13,14,15,17,18,22,23,24,25,32,37,38,44,45,46,47,51,52,55,60]
2	Balance [7,8,28,30,35,43,51,57]
3	Postural control/trunk control [7,8,27,28,35,57]
4	Visual attention [16]
5	Muscle activation [19]
6	Disability levels [20]
7	Gait [21,50,55]
8	Activities of daily living [22,30,34]
9	Mobility [24]
10	Self-care [25]
10	Communication/functional speech [29,34]
11	Feeding and swallowing [31]
12	Muscle tone [22,32,51]
13	Upper extremity function [33,41]
14	Quality of life [22,34]
15	Clinical parameters [36]
16	Lower limb muscle architecture [37]
17	Prevention of natural progression of hip dislocation [39]
18	Postural symmetry in sitting and standing [40]
19	Motor skill acquisition [41]
20	Functional performance [42]
21	Satisfaction of parents [42]
22	Ambulatory skills [46]
23	Muscle strength [51]
24	Pulmonary function [58]

**Table 2:** Summary of the outcome measures used in various studies observing the effect of NDT on children with CP NDT: neurodevelopmental treatment; CP: cerebral palsy

S. No	Outcome measure used
1	Gross motor function measure (GMFM) [7,8,10,11,12,13,17,18,22,23,24,25,27,28,32,37,38,44,46,47,51,52,55,60]
2	Postural assessment scale [7]
3	Trunk impairment scale [7,31,35]
4	Pediatric balance scale [7,8,35,52]
5	1-minute walking test [8]
6	Modified timed up-and-go test [8]
7	Functional Independence Measure for Children (WeeFIM) [8,22,30]
8	Seated postural control measurement [8,28,57]
9	Test of infant motor performance (TIMP) [14]
10	Angular movements of Hip, knee, and ankle joints using a 3D, four-camera analysis system [15]
11	Muscle activity using electromyography (EMG) [19,47]
12	Conners Kiddie Continuous Performance Test [16]
13	Gross motor functional classification system (GMFCS) [20]
14	Pedographs were used to measure gait including stride and step length, foot angle, base of support, cadence, and velocity [21,46]
15	The parent form of the child health questionnaire (CHQ-PF50) [22]
16	Pediatric evaluation of disability inventory (PEDI) [24,25,46]
17	Timed up and go test [24,30,34,52]
18	Kyphosis levels [27]
19	Questionnaire for dysarthria of Puyuelo [29]
20	Functional reach test [30]
21	Sit to stand test [30]
22	Nintendo Wii Fit balance [30]
23	Age and game score [30]
24	10-minute walk test [30]
25	10-step climbing test [30]
26	Schedule for oral motor assessment [31]
27	Pediatric quality of life inventory (PedsQL) [31,34]
28	Box and block test (BBT) [32]
29	Modified Ashworth scale [32,51]
30	Minnesota Manual Dexterity Test (MMDT) [33]
31	Childhood Health Assessment Questionnaire (CHAQ) [33]
32	Duruoz Hand Index (DEI) [33]
33	Dynamometer [33]
34	Communication function classification system [34]
35	Visual analogue scale [34]
36	Katz scale to measure ADLs [34]
37	Viking speech scale (VSS) [34]
38	Trunk control measurement scale [35,57]
39	Mobility questionnaire [37]
40	Goal Attainment Scale [36,38]
41	3D gait analysis [36]
42	Ultrasonography [37]
43	Mobility questionnaire (MobQue) [37]
44	Root mean square of head accelerations [38]
45	Hip radiographs to measure migration percentage [38]
46	Postural symmetry measured by the Boditrak pressure mapping system [40]
47	Peabody developmental motor scales-2^nd^ edition [41,45]
48	Quality of upper extremity skills test (QUEST) [41]
49	ACQUIRE therapy motor patterns and functional activities [41]
50	Berg balance scale [43]
51	Biomechanical evaluation performed using "Stabilometry footboard PoData 2.00" [43]
52	Timed 10-metre walk test [46]
53	Manual Muscle testing [51,57]
54	Spatiotemporal gait parameters [52]
55	Stair-climbing mechanical efficiency [55]
56	Parent questionnaire rating child mobility [55]
57	Pediatric reach test [57]
58	Pediatric berg balance measurement [57]
59	6-minute walking test [57]
60	Observational gait scale [57]
61	Core stability performance measurement [57]
62	Lung volumes [58]

## Conclusions

In our review, 19 studies suggested positive outcomes owing to NDT in children with CP; 22 studies supported the use of NDT in combination with other treatment methods; and 13 studies indicated uncertainty where authors had conflicting views about the use of NDT in children with CP. The review revealed that NDT is widely used for the rehabilitation of children with CP all over the world. The areas of improvement analyzed in the studies on NDT mostly involve gross motor, balance, and postural control. The parameters and outcome measures used in studies investigating the effect of NDT are mostly linked to body structure and function or activities according to the ICF model by WHO. There is a scarcity of published data available on the effect of NDT in terms of the participation component of ICF. There is scope for future research to analyze the effect of NDT in areas of improvement from a participatory perspective and how functional gain by the application of NDT is translated into participation for children with CP. Studies with larger sample sizes and homogenous groups along with a clear elaboration of NDT strategies should be conducted to gain deeper insights into the topic.

## References

[1] Ryan JM, Cassidy EE, Noorduyn SG, O'Connell NE (2017). Exercise interventions for cerebral palsy. Cochrane Database Syst Rev.

[2] Vyas AG, Kori VK, Rajagopala S, Patel KS (2013). Etiopathological study on cerebral palsy and its management by Shashtika Shali Pinda Sweda and Samvardhana Ghrita. Ayu.

[3] Peter Rosenbaum, Nigel Paneth, Alan Leviton (2023). UCP Educational and Research Foundation: A report: the definition and classification of cerebral palsy April 2006. UCP educational and research foundation.

[4] Patel DR, Neelakantan M, Pandher K, Merrick J (2020). Cerebral palsy in children: a clinical overview. Transl Pediatr.

[5] Nshimiyimana J, Uwihoreye P, Muhigirwa JC, Niyonsega T (2023). Neurofunctional intervention approaches. Neurorehabilitation and Physical Therapy.

[6] Howle JM, Neuro-Developmental Treatment Association Theory Committee (2004). Neuro-developmental Treatment Approach: Theoretical Foundations and Principles of Clinical Practice. Neuro-developmental Treatment Approach: Theoretical Foundations and Principles of Clinical Practice.

[7] Sah AK, Balaji GK, Agrahara S (2019). Effects of task-oriented activities based on neurodevelopmental therapy principles on trunk control, balance, and gross motor function in children with spastic diplegic cerebral palsy: a single-blinded randomized clinical trial. J Pediatr Neurosci.

[8] Tekin F, Kavlak E, Cavlak U, Altug F (2018). Effectiveness of neuro-developmental treatment (Bobath concept) on postural control and balance in cerebral palsied children. J Back Musculoskelet Rehabil.

[9] Franki I, Desloovere K, De Cat J (2012). The evidence-base for conceptual approaches and additional therapies targeting lower limb function in children with cerebral palsy: a systematic review using the ICF as a framework. J Rehabil Med.

[10] Lee KH, Park JW, Lee HJ, Nam KY, Park TJ, Kim HJ, Kwon BS (2017). Efficacy of intensive neurodevelopmental treatment for children with developmental delay, with or without cerebral palsy. Ann Rehabil Med.

[11] Tsorlakis N, Evaggelinou C, Grouios G, Tsorbatzoudis C (2004). Effect of intensive neurodevelopmental treatment in gross motor function of children with cerebral palsy. Dev Med Child Neurol.

[12] Swiggum MS, Knowlton J, Powers D (2021). Short-term and sustained effects of a three-week neuro-developmental treatment intensive: a case series report. NeuroRehabilitation.

[13] LA S, SH A, HO MT, SO V, Shakibaee A (2015). Effects of neurodevelopmental therapy on gross motor function in children with cerebral palsy. Iran J Child Neurol.

[14] Lee EJ (2017). Effect of neuro-development treatment on motor development in preterm infants. J Phys Ther Sci.

[15] Yonetsu R, Iwata A, Surya J, Unase K, Shimizu J (2015). Sit-to-stand movement changes in preschool-aged children with spastic diplegia following one neurodevelopmental treatment session--a pilot study. Disabil Rehabil.

[16] Abuin-Porras V, Pedersini P, Berjano P, Villafañe JH (2019). The efficacy of physical therapy on the improvement of the motor components of visual attention in children with cerebral palsy: a case series study. J Exerc Rehabil.

[17] Kalaichandran K, Swarnakumari DP (2019). Neuro developmental treatment (NDT) for cerebral palsy: - a clinical study. Int J Innov Res Sci Eng Technol.

[18] Senthilkumar CB, Deepa B, Ramadoss K (2009). Effect of neurodevelopmental therapy in gross motor function of children with cerebral palsy. Indian J Physiother Occup Ther.

[19] Paludo T, Zardo F, de Mattos BT, Frata B, Ling CC, de Castro Barroso G, Cechetti F (2023). Measuring muscle activation using electromyography during neurodevelopmental treatment in individuals with severe cerebral palsy. J Back Musculoskelet Rehabil.

[20] Ezema CI, Lamina S, Nkama RE, Ezugwu UA, Amaeze AA, Nwankwo MJ (2014). Effect of neuro-developmental therapy (NDT) on disability level of subjects with cerebral palsy receiving physiotherapy at the University of Nigeria Teaching Hospital, Enugu, Nigeria. Niger J Paediatr.

[21] Adams MA, Chandler LS, Schuhmann K (2000). Gait changes in children with cerebral palsy following a neurodevelopmental treatment course. Pediatr Phys Ther.

[22] Turker D, Korkem D, Ozal C, Gunel M, Karahan S (2015). Int J Ther Rehabil Res. The effects of neuro-developmental (Bobath) therapy based goal-directed.

[23] Desouzart G (2018). Physiotherapy intervention according to the Bobath concept in a clinical case of cerebral palsy. Orthop Res Online J.

[24] Besios T, Nikolaos A, Vassilios G, Giorgos M, Tzioumakis Y, Comoutos N (2018). Effects of the neurodevelopmental treatment (NDT) on the mobility of children with cerebral palsy. Open J Ther Rehabil.

[25] Knox V, Evans AL (2002). Evaluation of the functional effects of a course of Bobath therapy in children with cerebral palsy: a preliminary study. Dev Med Child Neurol.

[26] Araújo PA, Starling JM, Oliveira VC, Gontijo AP, Mancini MC (2020). Combining balance-training interventions with other active interventions may enhance effects on postural control in children and adolescents with cerebral palsy: a systematic review and meta-analysis. Braz J Phys Ther.

[27] Karabay İ, Doğan A, Ekiz T, Köseoğlu BF, Ersöz M (2016). Training postural control and sitting in children with cerebral palsy: Kinesio taping vs. neuromuscular electrical stimulation. Complement Ther Clin Pract.

[28] Elbasan B, Akaya KU, Akyuz M, Oskay D (2018). Effects of neuromuscular electrical stimulation and Kinesio Taping applications in children with cerebral palsy on postural control and sitting balance. J Back Musculoskelet Rehabil.

[29] Puyuelo M, Rondal JA (2005). Speech rehabilitation in 10 Spanish-speaking children with severe cerebral palsy: a 4-year longitudinal study. Pediatr Rehabil.

[30] Tarakci D, Ersoz Huseyinsinoglu B, Tarakci E, Razak Ozdincler A (2016). Effects of Nintendo Wii-Fit(®) video games on balance in children with mild cerebral palsy. Pediatr Int.

[31] Acar G, Ejraei N, Turkdoğan D, Enver N, Öztürk G, Aktaş G (2022). The effects of neurodevelopmental therapy on feeding and swallowing activities in children with cerebral palsy. Dysphagia.

[32] Salazar Fajardo JC, Kim R, Gao C, Hong J, Yang J, Wang D, Yoon B (2022). The effects of tDCS with NDT on the improvement of motor development in cerebral palsy. J Mot Behav.

[33] Avcil E, Tarakci D, Arman N, Tarakci E (2021). Upper extremity rehabilitation using video games in cerebral palsy: a randomized clinical trial. Acta Neurol Belg.

[34] Ejraei N, Ozer AY, Aydogdu O, Turkdogan D, Polat MG (2021). The effect of neck-trunk stabilization exercises in cerebral palsy: randomized controlled trial. Minerva Pediatr (Torino).

[35] Bilek F, Tekin F (2021). Effectiveness of extracorporeal shock wave therapy on postural control and balance in children with unilateral cerebral palsy: a randomized controlled trial. Percept Mot Skills.

[36] Desloovere K, De Cat J, Molenaers G (2012). The effect of different physiotherapy interventions in post-BTX-A treatment of children with cerebral palsy. Eur J Paediatr Neurol.

[37] Lee M, Ko Y, Shin MM, Lee W (2015). The effects of progressive functional training on lower limb muscle architecture and motor function in children with spastic cerebral palsy. J Phys Ther Sci.

[38] Tramontano M, Medici A, Iosa M, Chiariotti A, Fusillo G, Manzari L, Morelli D (2017). The effect of vestibular stimulation on motor functions of children with cerebral palsy. Motor Control.

[39] Picciolini O, LE Métayer M, Consonni D (2016). Can we prevent hip dislocation in children with cerebral palsy? Effects of postural management. Eur J Phys Rehabil Med.

[40] Holland H, Blazek K, Haynes MP, Dallman A (2019). Improving postural symmetry: The effectiveness of the CATCH (Combined Approach to Treatment for Children with Hemiplegia) protocol. J Pediatr Rehabil Med.

[41] Haynes MP, Phillips D (2012). Modified constraint induced movement therapy enhanced by a neuro-development treatment-based therapeutic handling protocol: two case studies. J Pediatr Rehabil Med.

[42] Kolit Z, Ekici G (2023). Effect of the Cognitive Orientation to daily Occupational Performance (CO-OP) approach for children with cerebral palsy: a randomized controlled trial. J Pediatr Rehabil Med.

[43] Ungureanu A, Rusu L, Rusu MR, Marin MI (2022). Balance rehabilitation approach by Bobath and Vojta methods in cerebral palsy: a pilot study. Children (Basel).

[44] Shamsoddini A (2010). Comparison between the effect of neurodevelopmental treatment and sensory integration therapy on gross motor function in children with cerebral palsy. Iran J Child Neurol.

[45] Behzadi F, Noroozi H, Mohamadi M (2014). The comparison of neurodevelopmental-Bobath approach with occupational therapy home program on gross motor function of children with cerebral palsy. J Rehabil Sci Res.

[46] Begnoche DM, Pitetti KH (2007). Effects of traditional treatment and partial body weight treadmill training on the motor skills of children with spastic cerebral palsy. A pilot study. Pediatr Phys Ther.

[47] Choi M, Lee D, Ro H (2011). Effect of task-oriented training and neurodevelopmental treatment on the sitting posture in children with cerebral palsy. J Phys Ther Sci.

[48] Dewar R, Love S, Johnston LM (2015). Exercise interventions improve postural control in children with cerebral palsy: a systematic review. Dev Med Child Neurol.

[49] Zanon MA, Pacheco RL, Latorraca CO, Martimbianco AL, Pachito DV, Riera R (2019). Neurodevelopmental treatment (Bobath) for children with cerebral palsy: a systematic review. J Child Neurol.

[50] Das SP, Ganesh GS (2019). Evidence-based approach to physical therapy in cerebral palsy. Indian J Orthop.

[51] Park EY, Kim WH (2017). Effect of neurodevelopmental treatment-based physical therapy on the change of muscle strength, spasticity, and gross motor function in children with spastic cerebral palsy. J Phys Ther Sci.

[52] Kim MR, Lee BH, Park DS (2016). Effects of combined Adeli suit and neurodevelopmental treatment in children with spastic cerebral palsy with gross motor function classification system levels I and II. Hong Kong Physiother J.

[53] Anttila H, Autti-Rämö I, Suoranta J, Mäkelä M, Malmivaara A (2008). Effectiveness of physical therapy interventions for children with cerebral palsy: a systematic review. BMC Pediatr.

[54] Harris SR, Roxborough L (2005). Efficacy and effectiveness of physical therapy in enhancing postural control in children with cerebral palsy. Neural Plast.

[55] Bar-Haim S, Harries N, Nammourah I (2010). Effectiveness of motor learning coaching in children with cerebral palsy: a randomized controlled trial. Clin Rehabil.

[56] Te Velde A, Morgan C, Finch-Edmondson M (2022). Neurodevelopmental therapy for cerebral palsy: a meta-analysis. Pediatrics.

[57] Adıguzel H, Elbasan B (2022). Effects of modified pilates on trunk, postural control, gait and balance in children with cerebral palsy: a single-blinded randomized controlled study. Acta Neurol Belg.

[58] Kanna BSS, Balabaskar K (2019). A study on efficacy of respiratory exercises coupled with neurodevelopmental treatment on pulmonary function of children with spastic quadriplegic cerebral palsy. Biomed Pharmacol J.

[59] Martin L, Baker R, Harvey A (2010). A systematic review of common physiotherapy interventions in school-aged children with cerebral palsy. Phys Occup Ther Pediatr.

[60] Khayatzadeh Mahani M, Karimloo M, Amirsalari S (2011). Effects of modified Adeli suit therapy on improvement of gross motor function in children with cerebral palsy. Hong Kong J Occup Ther.

[61] Barry MJ (1996). Physical therapy interventions for patients with movement disorders due to cerebral palsy. J Child Neurol.

[62] Furtado MA, Ayupe KM, Christovão IS, Sousa Junior RR, Rosenbaum P, Camargos AC, Leite HR (2022). Physical therapy in children with cerebral palsy in Brazil: a scoping review. Dev Med Child Neurol.

